# Tocopherol Profile Variations of Soybean World Mini-Core Collection Accessions Grown in Hokkaido

**DOI:** 10.17912/micropub.biology.001813

**Published:** 2026-01-27

**Authors:** Maria Stefanie Dwiyanti

**Affiliations:** 1 Research Faculty of Agriculture, Hokkaido University, Sapporo, 01, JP

## Abstract

Tocopherols (vitamin E) are lipid-soluble antioxidants, with α-tocopherol having the highest vitamin E activity. Soybean seed is a major source of tocopherols. Therefore, improving seed tocopherol content is a key breeding objective. Thirty-eight accessions from the soybean world mini-core collection were evaluated in 2019 and 2022 growing seasons. Four accessions carried a high α-tocopherol associated allele at S09_44341929 but they did not reach the high α-tocopherol levels of cultivar KAS, likely due to cooler seed-filling conditions. *Moshido Gong 502* and *503* showed low total tocopherol content in both seasons, making them promising resources for investigating genetic regulation of tocopherol biosynthesis.

**Figure 1. Variation in tocopherol content and flowering time among 38 soybean accessions:**
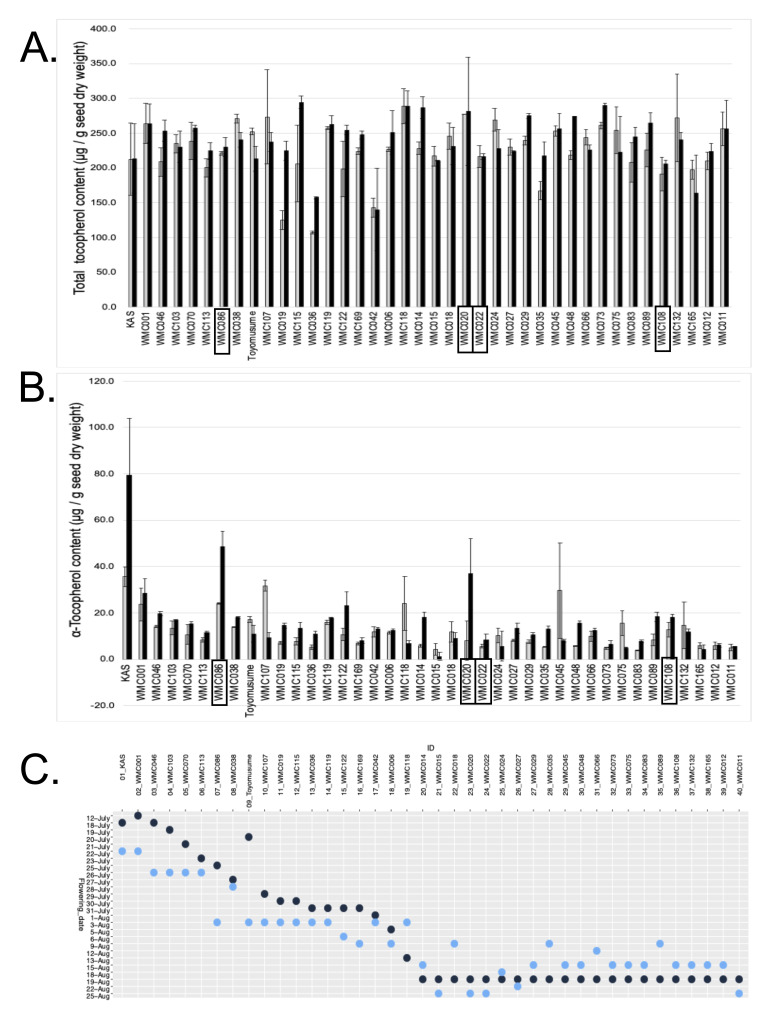
Variation in tocopherol content and flowering time among 38 soybean accessions.(A) Total tocopherol content of 38 accessions grown in 2019 (grey) and 2022 (black). (B) α-Tocopherol content of the same accessions in 2019 (grey) and 2022 (black). For (A) and (B), values represent the mean of two biological replicates, with error bars indicating standard deviation. Accessions having ‘A’ allele at SNP S09_44341929 are marked with rectangles.(C) Flowering time of each accession in 2019 (dark blue) and 2022 (light blue).

## Description

Tocopherols are lipid-soluble antioxidants belong to vitamin E group (Bramley et al. 2000). There are four isoforms of tocopherols: α-, β-, γ-, and δ-tocopherol, among which α-tocopherol showing the highest vitamin E activity. Soybean seeds contain γ-Toc (60-70% of total Toc content), followed by δ-Toc (20-30%), α-Toc (<10%), and β-Toc (1-2%) (Bramley et al *.* 2000). Increasing the proportion of α-tocopherol, along with maintaining high total tocopherol levels, would significantly enhance the nutritional value of soybean. Indeed, studies have been conducted to identify genetic regulation underlying Toc biosynthesis and to alter Toc composition and content through genetic engineering or conventional breeding (Van Eenennaam et al *.* 2003, Hagely et al. 2021).

The NARO Genebank World Soybean Core Collection, also known as the soybean World Mini-core Collection (WMC), contains 96 soybean accessions representing global genetic diversity Kaga et al. (2012). These accessions have been resequenced, and genome-wide SNP data are publicly available (Kajiya-Kanegae et al *.* 2021). This makes the collection a valuable resource to study natural variations in tocopherol content and composition. Due to Hokkaido short growing season, only 38 accessions reached seed maturity and could be harvested before snowfall. Nevertheless, these accessions provide useful insights into the regulation of tocopherol biosynthesis.

Previous studies identified genomic regions associated with soybean seed tocopherol composition through QTL analysis (Dwiyanti et al *.* 2011, Park et al. 2019). In particular, the α-tocopherol ratio in soybean seeds is primarily regulated by γ-tocopherol methyltransferase (γ-TMT). Soybean possesses three γ-TMT gene, one of which, *γ-TMT3* was identified as the causal gene conferring high α-tocopherol ratio in soybean variety Keszthelyi Aproszemu Sarga (KAS) (Dwiyanti et al. 2011) and in wild soybean B04009 (Park et al. 2019). Two SNPs located in the *γ-TMT3* promoter region, within MYBCORE and CAAT cis-elements, were associated with increased α-tocopherol ratio (Dwiyanti et al *.* 2011). Among them, SNP S09_44341929 (CAAT box) distinguished high α-tocopherol (‘A’ allele) from low α-tocopherol genotypes (‘C’ allele) (Dwiyanti et al *.* 2011, Park et al *.* 2019).

Based on the published SNP data (Kajiya-Kanegae et al *.* 2021), four WMC accessions (WMC020, WMC022, WMC108, and WMC086) carried ‘A’ allele at S09_44341929 ( Figure 1A, B). Despite this favorable allele, the α-tocopherol content of four accessions were lower compared to the previously identified high α-tocopherol cultivar KAS. It is known that α-tocopherol is highly affected by temperature during seeds maturation (Britz and Kremer 2002, Chennupati et al *.* 2011). KAS flowered on July 18 ^th^ , 2019 and July 22 ^nd^ , 2022 ( Figure 1C ). For 2019 growing season, WMC086 flowered on July 25 ^th^ , whereas WMC020, WMC022, and WMC108 flowered on August 18 ^th^ ( Figure 1C ). For 2022 growing season, WMC086, WMC020, WMC022, and WMC108 flowered on August 3 ^rd^ , August 22 ^nd^ , August 22 ^nd^ , and August 9 ^th^ , respectively ( Figure 1C ). The monthly minimum and maximum temperatures in 2019 growing season were 18.7 °C – 26.1 °C (July), 19.7 °C – 26.5 °C (August), 15.4 °C – 24 °C (September), and 8.9 °C – 17.5 °C (October). Corresponding minimum and maximum temperatures in 2022 growing season were 20 °C – 27.3 °C (July), 19.5 °C – 26.8 °C (August), 15.6 °C – 24.1 °C (September), and 8.4 °C – 17.3 °C (October). Because seed filling in the four WMC accessions occurred at lower temperature than in KAS, this likely contributed to their lower α-tocopherol content.

Total tocopherol content of 38 WMC accessions ranged from 107.2 μg/g seed dry weight to 288.9 μg/g seed dry weight in 2019, and 140.1 μg/g seed dry weight to 289.9 μg/g seed dry weight in 2022 ( Figure 1A ). Total tocopherol content was not correlated with flowering time ( Figure 1A, 1C). WMC036 (MoshidoGong 502) and WMC042 (MoshidoGong 503) consistently exhibited lower total tocopherol content compared to other WMC accessions. These accessions,characterized by smaller seeds and semi-wild phenotypes (Chen and Nelson 2004, Kaga et al *.* 2012), represent promising materials for further genetic dissection. QTL analysis using segregating populations derived from these accessions may reveal novel loci regulating total tocopherol content.

## Methods

**Plant materials and growing condition**

Thirty-eight early flowering accessions from WMC were used in this study. For comparison, a low α-tocopherol Hokkaido cultivar Toyomusume and high α-tocopherol cultivar KAS were included. Four plants per accession were grown in summer 2019, and six plants per accession were grown in summer 2022 at the experimental farm of Hokkaido University (43°4’N, 141°20’E). The experimental farm was rain watered. The monthly minimum and maximum temperatures in 2019 growing season and 2022 growing season (May to November) was obtained from Japan Meteorological Agency, by selecting Sapporo as specific location. Flowering time was recorded as the date of first flower appearance. Seeds were harvested in bulk, dried, and stored at 4 °C until analysis.

&nbsp;

**Tocopherol analysis**

Tocopherol extraction and quantification were performed following procedure described in Dwiyanti et al *.* (2011). Five seeds per replication were ground into fine powder using a multi-bead shocker (Yasui Kikai, Japan; 3,000 rpm, 10 s, three cycles). Fifty milligrams of powder was saponified in 500 μl of 80% ethanol containing internal standard dl-tocol (Tama Biochemical, Japan) by vortexing and sonicating the samples for 10 min at room temperature. Subsequently, 1 ml of hexane was added to sample and the mixture was sonicated for 10 min at room temperature. After centrifugation (13,000 rpm, 5 minutes), 600 μl of the upper hexane layer was transferred to a HPLC vial. HPLC analysis was performed using Inertsil ODS-3 column (30 mm × 250 mm, GL Sciences, Japan) at 40 °C, with acetonitrile:methanol (9:1, v/v) as the mobile phase at flow rate 0.5 mL/min. Sample injection volume was 30 μL. Tocopherol peaks were detected using 295 nm UV light. The analysis method used in this study could not separate γ-tocopherol and β-tocopherol, but the amount of β-tocopherol in soybean seeds is negligible, thus γ-tocopherol and β-tocopherol were calculated as γ-tocopherol. Measurements from each accession were averaged and reported. Replicates were averaged and reported per accession.

&nbsp;

**SNP data information**

All accessions’ genotype information of SNP S09_44341929 (rs124692153), located in the promoter region of *γ-TMT3* gene (Glyma.09G222800) were obtained from the the multiple genome browser TASUKE ( https://daizutasuke275-core.daizu.dna.affrc.go.jp/ ) (last accessed on 18 December 2023, Kajiya-Kanegae et al. 2021). The position of SNPs in TASUKE browser was based on reference genome Williams82.v2.a1. SNPs were filtered using the following criteria:variant quality ≥ 20, total read depth ≥ 2, and depth of alternative alleles ≥ 2. The genome browser was moved to DAIZU-NET and the positions were based on the reference genome Williams82.v4.a1 (https://daizu-net.dna.naro.go.jp/ap/reseq467woDP-wm82v4,

last accessed December 18 ^th^ , 2025). SNP S09_44341929 position was based on the soybean reference genome Williams82.v2.a1, and based on the reference genome Williams82.v4.a1, it is located on Chromosome 09: 45,209,951.
